# Decorin reduces white matter pathology in experimental hydrocephalus: a diffusion tensor imaging and immunohistochemical study

**DOI:** 10.1186/2045-8118-12-S1-O59

**Published:** 2015-09-18

**Authors:** James Patterson McAllister, Anuriti Aojula, Hannah Botfield, Osama Abdullah, Ana Maria Gonzalez, Dustin Ragan, Ann Logan, Alexandra Sinclair

**Affiliations:** 1 Neurosurgery, Washington University School of Medicine, USA; 2 Neurotrauma and Neurodegeneration, School of Clinical and Experimental Medicine, University of Birmingham, UK; 3 Bioengineering, University of Utah, USA

## Introduction

We have shown previously that Decorin, by antagonizing TGF-β–mediated subarachnoid fibrosis, prevents ventriculomegaly in experimental juvenile hydrocephalus. To focus on white matter alterations, we sought to correlate cytopathological changes induced by hydrocephalus with diffusion tensor imaging (DTI) parameters and determine if Decorin could prevent these changes.

## Methods

Communicating hydrocephalus was induced in 3-week-old rats with basal cistern injections of kaolin; age-matched controls were intact (n=4) and kaolin-no treatment (n=4) animals. Immediately following kaolin injections, animals received a 14-day continuous intraventricular infusion of phosphate-buffered saline (n=6) or human recombinant Decorin (n=5) via osmotic minipumps. At 14 days post-kaolin, all rats underwent MRI/DTI scanning followed immediately by sacrifice and brain fixation. DTI voxel-based analysis was performed on 4 serial rostral-to-caudal slices to quantify mean fractional anisotropy (FA), diffusivity (MD), axial diffusivity (AD) and radial diffusivity (RD) of the corpus callosum (CC) and periventricular white matter (PVWM). Immunohistochemistry and stereology were employed to quantify astrogliosis (GFAP) and aquaporin-4 (AQP4) levels in the CC and PVWM at caudal levels.

## Results

Compared to intact animals (rostral 1.3±0.1 and caudal 0.9±0.1 ventricular volume), the caudal lateral ventricles were significantly larger in kaolin-only (16.2±2.8, p=0.005) and kaolin-PBS (21.0±5.4,p<0.001) animals than rostral portions (8.0±1.7 and 10.1±3.8, respectively). Following this gradient, untreated hydrocephalic rats exhibited significantly (p<0.01) decreased FA and increased RD in the caudal-most CC and increased MD and AD in the caudal PVWM compared to intact controls. Decorin significantly (p<0.05) reversed the RD and MD changes in the caudal CC and PVWM MD (p<0.05). Such DTI reversals were not discovered in the rostral CC and PVWM. A significant increase in GFAP immunostaining resulted in a positive correlation (p<0.05) between CC GFAP levels and the caudal-most CC RD. In the caudal PVWM, MD and AQP4 levels and AD and GFAP presence were positively correlated (p<0.01).

## Conclusions

These results indicate that regional differences exist in ventricular and DTI parameters, and that Decorin has the therapeutic potential to decrease microstructural damage in juvenile hydrocephalus.
